# *Eriobotrya japonica* Water Extract Characterization: An Inducer of Interferon-Gamma Production Mainly by the JAK-STAT Pathway

**DOI:** 10.3390/molecules21060722

**Published:** 2016-06-02

**Authors:** Khalid Z. Matalka, Nada A. Abdulridha, Mujtaba M. Badr, Kenza Mansoor, Nidal A. Qinna, Fadi Qadan

**Affiliations:** 1 Department of Pharmacology and Biomedical Sciences, Faculty of Pharmacy and Medical Sciences, University of Petra, Amman 11196, Jordan; nido892@yahoo.com (N.A.A.); mujtabambadr@gmail.com (M.M.B.); nqinna@uop.edu.jo (N.A.Q.); 2 Petra University Pharmaceutical Center (PUPC), University of Petra, Amman 11196, Jordan; 3 Department of Pharmaceutical Medicinal Chemistry and Pharmacognosy, Faculty of Pharmacy and Medical Sciences, University of Petra, Amman 11196, Jordan; kmansoor@uop.edu.jo; 4 Eusano GmbH & Co., Tussenhausen 86874, Germany; fqadan@googlemail.com

**Keywords:** *Eriobotrya japonica*, IFN-γ, IL-12, immunomodulator, JAK-STAT, MALDI-TOF, MCA fibrosarcoma

## Abstract

*Eriobotrya japonica* (Thunb.) Lindl. (Loquat) (EJ) has been used as a medicinal plant to treat chronic bronchitis, coughs, phlegm, high fever and gastro-enteric disorders. Since the traditional use of EJ is related to modulating inflammation processes, our earlier studies on EJ leaves were performed on the water extract to investigate specific cytokines’ modulation. These earlier studies, however, have shown that EJ leaf water extract (WE) and the water phase (WP) induce cytokines’ production in *in vitro* and *in vivo* models. Therefore, the aim of this study was to specify the group(s) of compounds in EJ leaves that have this immunomodulatory activity and their mechanism of action. WE was obtained from boiling the leaves followed by butanol extraction, yielding a butanol-water phase (WP). WP was then subjected to methanol:acetone fractionation, yielding upper (MAU) and lower (MAL) phases. For further fractionation, MAU was subjected to column chromatography followed by elution with ethanol:water (EW), methanol:ethanol (ME) and, lastly, acetone:water (AW), respectively, to reveal three sub-fractions; MAU-EW, MAU-ME and MAU-AW. MAU-AW significantly increased IFN-γ production from unstimulated and stimulated mouse spleen cells, as well as CD3+ T cells and natural killer cells. Furthermore, the fold increase of IFN-γ production by MAU-AW was concentration dependent, higher than the parent extract or any of the other sub-fractions, and such an IFN-γ increase was reversed by two JAK-STAT inhibitors. In addition, MALDI-TOF-MS analysis of the extracts and sub-fractions showed compounds with molecular weights of >500 Daltons. The MAU-AW sub-fraction contained more polar compounds, such as flavonol and caffeic glycosides. In conclusion, these polar compounds in the EJ extract are responsible for inducing IFN-γ production. Further chemical elucidation is warranted to lead to a specific IFN-γ inducer and an immunomodulator in polarizing immune cells and balancing immune responses in certain diseases.

## 1. Introduction

*Eriobotrya japonica* (Thunb.) Lindl. (Loquat) (EJ) is a member of the Rosaceae family and has been used as a medicinal plant in China and Japan since ancient times. All parts of the plant are traditionally used for different ailments; e.g., pharyngolaryngitis, nosebleed, cough, bronchitis, constipation, diarrhea, depression and skin diseases [1]. The astringent leaves of EJ have been used for a long time to treat chronic bronchitis, coughs, phlegm, high fever and gastro-enteric disorders [2]. In Jordan, it has a long history of use in traditional medicine to treat diabetes [3,4]. Such traditional usage encouraged researchers to study the pharmacological effect of EJ. For instance, previous studies on the triterpene acids isolated from EJ showed a significant anti-viral [5], anti-inflammatory [5,6,7,8,9,10], anti-mutagenic [11] and anti-tumor properties [12,13,14]. 

Since the traditional use of EJ is related to modulating inflammation processes, our earlier studies on EJ leaves were performed on the water extract to investigate specific cytokines’ modulation. These earlier studies have shown that EJ leaf water extract (WE) induces cytokines’ production from unstimulated human blood cells in a dose-dependent manner. The response was in favor of pro-inflammatory cytokines’ production, such as interleukin (IL)-12, interferon (IFN)-γ and tumor necrosis factor-alpha (TNF)-α, more than an anti-inflammatory cytokine, IL-10 [15]. Further fractionation of WE with butanol resulted in a water-phase termed WP that produced higher amounts of pro-inflammatory cytokines *in vitro* [15] and *in vivo* [16]. WP also modulated cytokines toward IFN-γ in the tumor microenvironment and enhanced the survival of mouse bearing fibrosarcoma [16]. Such pharmacological behavior reflects the type of initial extraction and the solvent used. Similarly, Sun *et al.* showed that oral administration of EJ seed extracts in rats decreased IL-4 in the allergic dermatitis lesion and elevated IFN-γ, IL-2 and IL-10 levels, suggesting balancing of T helper 1 and 2 cytokines’ response in allergic dermatitis lesions [17,18]. 

The aim of this study is to specify the groups of compounds from the WP extract that are causing this specific cytokine induction. Therefore, further fractionation of the WP was performed in order to identify the sub-fractions and their constituents that have this immunomodulatory activity. The long-term goal, however, is finding an immunomodulator that can polarize immune cells and balance immune responses in T helper 2 immune-mediated diseases, such as allergies and systemic lupus erythematosus. 

## 2. Results

### 2.1. Different EJ Extracts Stimulated IFN-γ Production Partially through IL-12 from Mouse Spleen Cells

WP, MAU and MAL significantly increased IFN-γ production from un-stimulated and phytohemagglutinin (PHA) + LPS-stimulated mouse spleen cells (Table 1). However, the fold increase of IFN-γ production by MAU was concentration dependent and higher than the parent extract or its residue (Table 1). To test if this increase in IFN-γ production was dependent (or not) on IL-12 pathway from macrophages, extracts were cultured with isolated macrophages. All extracts showed a trend of the fold increase of IL-12 produced, but without reaching significant levels, suggesting that EJ extracts partially induce IFN-γ production via the IL-12 pathway (Table 1).

Furthermore, to investigate if EJ extracts can induce IFN-γ production from T or NK cells without interaction or cytokines being released (e.g., IL-12) from other immune cells (e.g., macrophages), IFN-γ production was measured following the exposure of EJ extracts to isolated T and NK cells. WP and MAU extracts significantly increased IFN-γ production from mouse T cells in a concentration-dependent manner (Table 1). As for NK cells, MAU increased IFN-γ production at 1 and 10 μg/mL, whereas MAL increased IFN-γ only at 100 μg/mL (*p* < 0.05).

**Figure 1:**
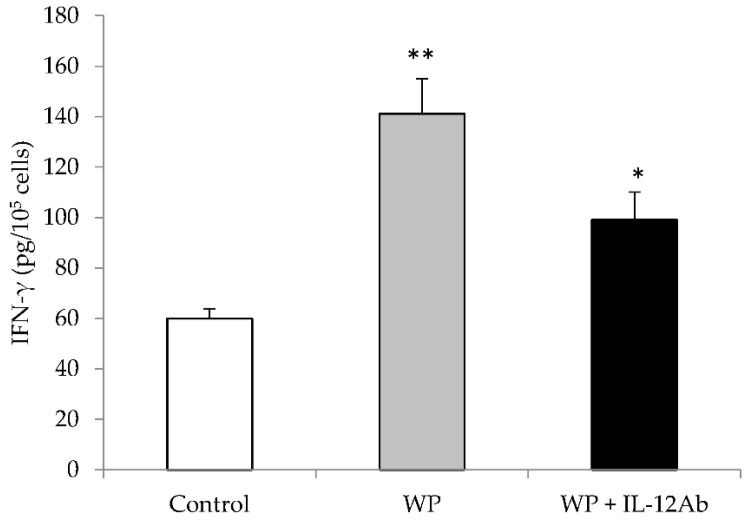
Anti-IL-12 antibody partially reversed the WP effect on IFN-γ production from mouse splenocytes. Adding anti-IL-12 antibody partially reduced the effect of WP on IFN-γ production from mouse splenocytes, but was not statistically significant. Data represent the average cytokine level from two independent experiments (±SEM), and each experiment consists of a minimum of six replicates per condition (* *p* < 0.05 and ** *p* < 0.01 compared to its zero counterpart).

Lastly, to confirm that IL-12-induced production by WP and/or its fractions partially induce IFN-γ production, anti-IL-12 antibodies (160 ng/mL) with WP were added to the spleen cell culture. Anti-IL-12 Ab partially reduced the effect of WP on IFN-γ levels, but did not block it completely (Figure 1).

### 2.2. MAU-AW Sub-Fraction Stimulated IFN-γ Production Better than the MAU-EW or MAU-ME Sub-Fractions from Unstimulated and Stimulated Mouse Spleen Cells

Since the MAU yields the best results regarding IFN-γ production, further sub-fractionation using column chromatography was performed. The first sub-fraction was with ethanol-water (MAU-EW), followed by methanol-ethanol (MAU-ME) and lastly by the acetone-water mixture (MAU-AW). All fractions induced a higher fold of IFN-γ than the parent extract WP from unstimulated and stimulated spleen cells (Table 2). In addition, the highest fold of IFN-γ produced was from the unstimulated spleen cells treated with MAU-AW. This fold increase, however, was independent on the concentrations used, 1 or 10 µg/mL (Table 2).

Similarly, to show that the EJ sub-fractions’ increase of IFN-γ production is partially related to IL-12 production, IL-12 was also measured following exposure to the sub-fractions. There was a relative fold increase in IL-12 in the unstimulated spleen cells treated with the sub-fractions. This increase was only significant for the MAU-AW sub-fraction (*p* < 0.001). When spleen cells were stimulated, almost all of the sub-fractions yield a significant production of IL-12 (Table 2). Furthermore, equal amounts of MAU sub-fractions were mixed together in order to investigate if there is an additive or inhibitory response on IFN-γ production from spleen cells. In general, the combinations of two fractions or all produced significant IFN-γ, but less than either one alone (data not shown). However, IL-12 production was similar to each sub-fraction alone. 

### 2.3. MAU-AW Sub-Fraction Induces IFN-γ Production Mainly by JAK-STAT 

**Figure 2:**
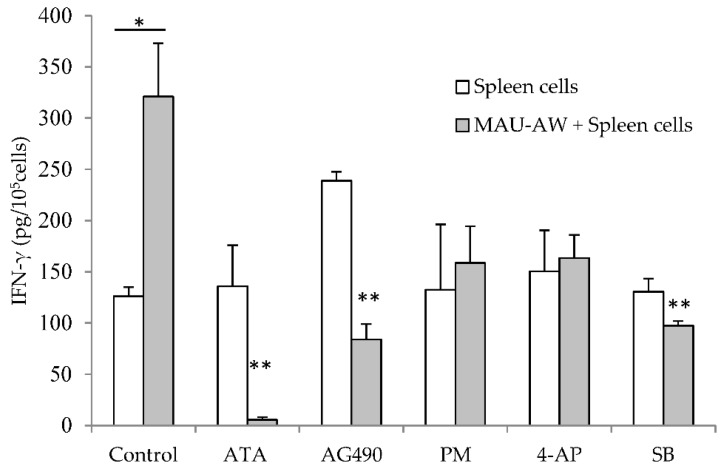
MAU-AW induces IFN-γ production mainly through the JAK-STAT pathway. MAU-AW increased IFN-γ production from mouse spleen cells (* *p* < 0.05). Adding ATA (50 µM), AG490 (50 μM) and SB (10 µM), but not PM (5 μM) nor 4-AP (5 mM), to the spleen cell culture for one hour prior to the addition of MAU-AW (10 µg/mL) reversed MAU-AW effect on IFN-γ production (** *p* < 0.01). Data represent the average of the cytokine level from two independent experiments (±SEM), and each experiment consists of a minimum of four replicates per condition.

To investigate the pathways responsible for the MAU-AW sub-fraction effect on IFN-γ, different pathways inhibitors were used to specify which pathway is responsible. Aurintricarboxylic acid (ATA) and tyrphostin AG 490 (AG490) act as inhibitors of the JAK-STAT signaling pathway; perhexiline maleate (PM) and 4-aminopyridine (4-AP) are potassium channel (Kv) blockers; and SB-203580 (SB) is a p38 MAPK inhibitor. The significant increase in IFN-γ production induced by the MAU-AW sub-fraction was reversed by ATA, AG490 and SB (Figure 2).

### 2.4. EJ Extract and Sub-Fractions Are Not Toxic to Mouse Spleen Cells

**Figure 3:**
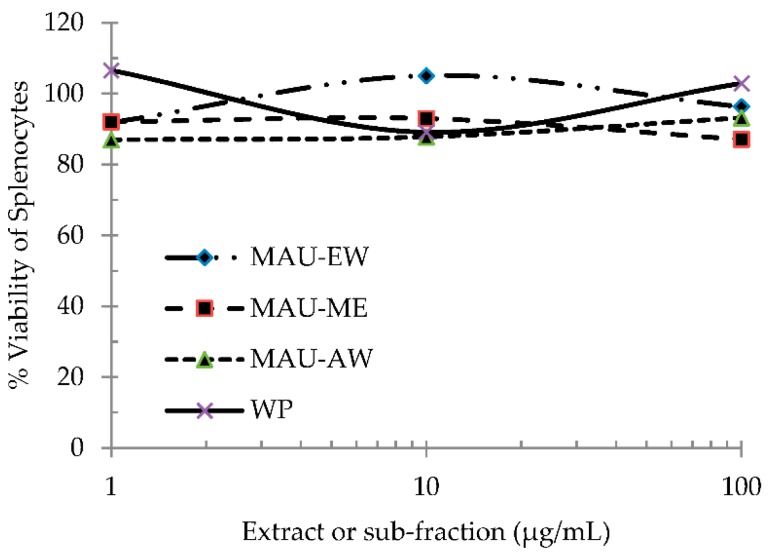
Cytotoxicity of WP, MAU-EW, MAU-ME and MAU-AW on mouse splenocytes. The MultiTox assay was performed following 48 h of incubation of several concentrations of each fraction with mouse spleen cells. At the end of incubation, live cell and dead cell substrates were added to all wells, mixed briefly on an orbital shaker and incubated for 30 min at 37 °C. The fluorescence was measured by the GloMax^®^-Multi Detection System for viability (excitation 495 nm; emission 505 nm), and (cytotoxicity: excitation 510 nm; emission 570 nm). Data represent the average of six replicates (±SEM) of the fluorescence signals of the alive/dead signals of each well. No reduction in % viability of spleen cells was observed.

The cytotoxicity of each extract and sub-fractions, WP, MAU-EW, MAU-ME and MAU-AW, on mouse spleen cells was tested. No significant effect on the % viability was observed by any of the four extracts (Figure 3).

### 2.5. MAU-AW Sub-Fraction Increased the IFN-γ Level in the MCA-Tumor Microenvironment 

**Figure 4:**
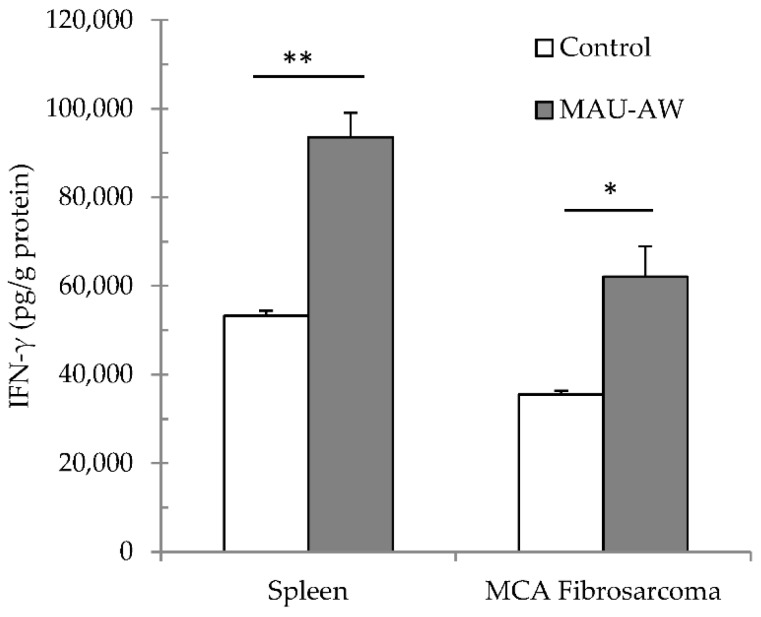
Cytokine modulation in spleen and MCA fibrosarcoma-bearing mice. Following three successive days of i.p. administration of MAU-AW (10 µg) in tumor-bearing mice, spleen and MCA fibrosarcoma were isolated, and IFN-γ levels were assayed in these tissues. MAU-AW increased IFN-γ levels in MCA fibrosarcoma, as well as spleen. Each data point represents four mice ± SEM (* *p* < 0.05; ** *p* < 0.01 *vs.* control tissue).

In order to confirm our hypothesis that the MAU-AW sub-fraction can increase IFN-γ in the tumor microenvironment, the MAU-AW sub-fraction (10 µg) was administered intra-peritoneally for three successive days into mice bearing MCA fibrosarcoma. Such administration significantly increased IFN-γ in the tumor microenvironment (*p* < 0.05, Figure 4). In addition, spleen IFN-γ levels were higher following such administration than those of control spleen counterparts (*p* < 0.01).

### 2.6. MALDI-TOF-MS Analysis

MALDI-TOF-MS analysis of EJ water extract and the sub-fractions showed the presence of known compounds in addition to some unidentified molecules, all of which have molecular weights greater than 500 Daltons (Figure S1 in the Supplementary Materials). WE contained flavonol glycosides, flavan-4-one glycoside, higher oligomeric procyanidins (A and B types) and cinchonain derivatives in glycosidic form. The WP, on the other hand, contained more polar compounds in comparison to both ethanolic and methanolic sub-fractions. The major identified compounds were oligomeric procyanidins, flavonol glycosides triterpene esters and cinnamic acid glycosides (Table 3).

The MAU-EW sub-fraction mainly contains the less polar compounds, like lower oligomeric procyanidins in addition to some flavan-4 one-glycosides, flavonol glycosides ursolic acid derivatives and cinchonain glycosides. On the other hand, MAU-AW sub-fractions contained more polar compounds in comparison to the MAU-EW and MAU-ME sub-fractions, and these were flavonol and caffeic acid glycosides (Table 3).

## 3. Discussion

A recent study has indicated that EJ water extract (WE) exhibits a significant immunomodulatory effect by inducing IL-12, TNF-α and IFN-γ [15]. Such an effect was thought to be related to the presence of polysaccharides and polar phenolic compounds, like procyanidins and flavonoid glycosides, in the extract. Extraction of HE by n-butanol to yield EJ water residue (WP) was previously performed to concentrate polysaccharides, high molecular weight oligomeric procyanidins and related polyphenols and to exclude low molecular weight compounds [12,16]. This WP extract was proven to induce the release of IFN-γ from mouse spleen cells and NK cells [16,29]. In the current investigation, however, further fractionation of WP was performed using methanol:acetone to yield the MAU fraction and MAL. The MAU fraction induced IFN-γ production from unstimulated, as well as PMA-stimulated mouse spleen cells better than the original extract, WP and MAL fraction, suggesting that the chemical compounds, such as polyphenols, tannins and flavonoids, were possibly the ones that could be responsible for the immunomodulatory effects of EJ leaves [12,23].

To understand such a mechanism, the extracts were tested on isolated T cells, NK cells and macrophages. Both MAU and MAL fractions significantly enhanced IFN-γ production from T and NK cells, with better enhancement shown by the MAU fraction. These effects were less effective on IL-12 production when the extracts were tested on isolated macrophages. In addition, when the anti-IL-12 antibody was added to cultures of splenocytes simultaneously with the addition of WP, IFN-γ induction levels were partially affected. This led to the conclusion that WP extract induces IFN-γ partially through the IL-12-mediated pathway. However, further fractionation with methanol:acetone reduced the IL-12-mediated pathway, but increased the IFN-γ activation pathway. Furthermore, JAK-STAT inhibitors reversed and blocked MAU-AW sub-fraction-induced IFN-γ production, indicating that JAK-STAT is the main signaling pathway of MAU-AW [30].

Most of the *Eriobotrya* extract-related studies revealed the anti-inflammatory action of such extracts. The anti-inflammatory action is mediated mainly by the triterpene acids and epicatechin isolated from *Eriobotrya* leaves [6,7,9,14,31]. However, studies by us and another group showed that EJ leaves polarize T helper 1 cytokines over T helper 2 cytokines [15,16,17,18,29]. Therefore, to identify the compounds or group of compounds that are responsible for the induction of IFN-γ, further sub-fractionation of MAU fraction was conducted by column chromatography using eluents with different polarities. The highest sub-fraction inducing IFN-γ production from unstimulated mouse spleen cells was by the acetone followed by methanol and, lastly, the ethanol sub-fraction. This indicates that the more polar compounds in the HE, as in the MAU-AW sub-fraction, are better at inducing IFN-γ. Furthermore, the MADLI-TOF analysis revealed compounds of more than 500 Daltons in each of the extracts and sub-fractions, indicating that the water extract does not contain the known anti-inflammatory compounds, triterpene acids and epicatechin [31]. 

While comparing our MALDI-TOF results to the literature on EJ, naringenin-8-C rhamnoglucoside, quercetin 3-sambubioside and cinchonain glucoside were interpreted to be present in all of the sub-fractions [12,23,24]. In addition and except for MAU-EW, quercetin-3-*O*-glucosyl-rhamnosyl-glucoside was also identified [23,24]. Furthermore, nerolidol 3-*O*-rhamnopyranosyl glucopyranoside was only identified in MAU-AW [7,26,27]. Furthermore and to the authors’ knowledge, very limited studies pointed out cytokines’ induction or modulation by the EJ constituents revealed herein. Amongst these studies, Mackenzie *et al.* pointed out that type-A dimeric procyanidin inhibits NF-kB and, thus, might have an anti-inflammatory activity [32,33]. In addition, gallic acid, which is a constituent of some of the *Eriobotrya* compounds, inhibited T helper-2 cytokines’ production (IL-4, IL-5 and IL-13) [34,35]. Therefore, it would be difficult to further interpret our results based on the data shown. However, it can be speculated that the compounds identified in MAU-AW (see above) are responsible for the IFN induction. 

In conclusion, further fractionation of WP extract and the concentration of active constituents induced better IFN-γ production from mouse spleen cells. Fractionating WP extract with methanol:acetone enhanced IFN-γ production from both stimulated and unstimulated spleen cells, isolated NK and T cells. This induction was partially mediated by the IL-12-IFN-γ pathway. Such induction was inhibited completely by JAK-STAT inhibitors. Since these EJ sub-fractions were not toxic to spleen cells and increase the production of IFN-γ, further chemical elucidation is warranted to lead to a specific IFN-γ inducer that will have a clinical potential as an immunomodulator. 

## 4. Materials and Methods

### 4.1. Chemicals and Reagents

RPMI-1640 media supplemented with fetal bovine serum (FBS), trypsin, penicillin/streptomycin and amphotericin B were obtained from Biochrom AB (Berlin, Germany). Aurintricarboxylic acid (ATA), tyrphostin AG 490 (AG490), perhexiline maleate (PM), 4-aminopyridine (4-AP), SB-203580 (SB), PD169316 (PD), Igepal CA-630, phytohemagglutinin (PHA) and lipopolysaccharide (LPS, L-6143) were obtained from Sigma (St. Louis, MO, USA). The mouse erythrocytes lysing kit was purchased from R&D systems (Minneapolis, MN, USA). Endotoxin-free Dulbecco’s PBS without calcium and magnesium were obtained from EuroClone S.P.A. (Siziano, Italy). Tissue culture 96-well flat bottom plates were purchased from Nalge Nunc International (Rochester, NY, USA).

### 4.2. Plant Material

Fresh EJ leaves were collected from a cultivated garden in the Tarek area (East Amman, Jordan; 9/2013), dried for 10 days at room temperature and identified in comparison with authentic EJ obtained from the Botanical Institute, University of Cologne (Cologne, Germany) and deposited under UOP039013. 

### 4.3. Plant Material Extraction

One hundred and thirty seven (137) grams of dried leaves were crushed by hand into small pieces, washed and extracted three times using 2 L of boiling distilled water for five minutes, then filtered. This extraction process yielded 1700 mL of EJ water extract (WE). One thousand four hundred fifty milliliters of WE were partitioned three times with butanol in a 1:1 ratio (*v*:*v*). The aqueous phase was collected to yield a 625-mL EJ water phase (WP). Three hundred seventy five milliliters of WP were partitioned three times with the same volumes of the methanol:acetone (7:3) mixture to yield two phases: the lower (MAL) and the upper (MAU). A 250-mL volume of each extract and sub-fraction was freeze dried, and the percentage of yield was calculated.

### 4.4. Column Chromatography

Five grams of MAU dissolved in ethanol were subjected to column chromatography (130 cm length and 5 cm width) on Sephadex^®^ LH-20 using 1.5 L of each of the following eluting solvent systems: ethanol:water (70:30), methanol:ethanol (50:50) and acetone:water (70:30) [3]. The collected sub-fractions were named MAU-EW, MAU-ME and MAU-AW, respectively. The three sub-fractions were then subjected to volume reduction by a rotary vacuum evaporator followed by freeze drying.

### 4.5. Animals

Adult female C57Bl/6 mice were obtained from Taconic Farms Inc. (New York, NY, USA) and housed at the University of Petra’s Animal House facility. All animal experiments were performed in compliance with FELASA guidelines (Federation of European Laboratory Animal Science Association), and the study protocol was approved by the Deanship of Scientific Research at the University of Petra (10/3/2013).

### 4.6. Mouse Spleen Cells Culture and Cytokines Analysis

Spleen tissues from mice were squeezed between two slides forming a suspension in RPMI culture media, centrifuged for 10 min, diluted with lysing buffer (2 mL) and incubated for 10 min at room temperature. The tube was filled with diluted washing buffer, centrifuged for 10 min and re-suspended in culture media. Spleen cells was adjusted by dilution to yield 10^6^ cells/mL, placed (10^5^/100 µL) into a sterile 96-well culture plates and incubated at 37 °C and 5% CO_2_ in a humidified incubator for 1 h before adding the extracts. Each extract was dissolved in culture media, sterilized by filtration with sterile filters (0.2 μm) and diluted to the required concentration. The plate was incubated for 48 h. In the stimulation culture experiments, a volume of 20 μL/well containing LPS and PHA at concentrations of 1 and 5 μg/mL, respectively, was added after 24 h [36]. The well contents were collected in 1-mL Eppendorfs followed by the addition of 50 μL of 0.1% igepal, incubated for 10 min and then stored at −30 °C. Cytokine (IFN-γ, IL-12) levels were analyzed using DuoSet ELISA development kits acquired from R&D Systems (Minnesota, MN, USA). Maxisorb 96-well flat bottom plates were purchased from Nalge Nunc International (Rochester, NY, USA). In addition, a rat monoclonal anti-mouse IL-12 p70 (IgG1) was acquired from R&D Systems. At the end of the ELISA immunoassay, the absorbance was read at 450 nm and 600 nm by the GloMax^®^-Multi Detection System (Promega Corporation, Madison, WI, USA).

### 4.7. Macrophage Cells Isolation

A volume of 15 mL of spleen cells suspension was added to 75 cm^2^ tissue culture flask, incubated for 1 h at 37 °C and 5% CO_2_ in a humidified incubator (Wahl and Smith, 2011) [37]. The non-adherent cells were decanted, and the flask was washed twice with 10 mL RPMI-1460 media to get rid of any residual non-adherent cells. The adherent macrophage cells were collected by scrapping and re-suspended in RPMI.

### 4.8. NK and T Cells Isolation

MagCellect Magnet, mouse CD3+ T cell isolation kit and MagCellect mouse NK cell isolation kit were obtained from R&D Systems. As the manufacturer recommendation, the procedures for isolating T and NK cells were followed. Collected cells were re-suspended in culture media and adjusted to the desired concentration for further applications.

### 4.9. MCA-Induced Tumors, Cell Lines Preparation and Inoculation

Mice (2–4 weeks old) were inoculated subcutaneously (s.c.) into the right hind flank with 1 mg/mouse of MCA dissolved in olive oil. Mice were inspected weekly for tumor development. When tumors reached 1–2 cm in diameter, mice were injected intra-peritoneally with MAU-AW (10 µg) for three successive days [16]. Twenty four hours post the last injection, mice were sacrificed, and tumor and spleen tissues were collected, weighed, placed into a pre-chilled tube and incubated with 2 mL of ice-cold endotoxin-free PBS containing 0.1% Igepal CA-630 under ice [36]. The tissues were then homogenized with a tissue disrupter (Janke and Kundle) and centrifuged (6000 rpm for 6 min), and the supernatant was transferred to labeled microcentrifuge tubes and stored at −30 °C until the cytokine assays.

### 4.10. MultiTox-Fluor Multiplex Cytotoxicity Assay

The spleen cell suspension at a concentration of 5 × 10^5^ was added to a 96-well plate with a black base (Corning Costar, New York, NY, USA) at volumes of 100μL/ well and incubated for 1 h. A volume of 100 μL culture media was added to the control wells and 75 μL culture media to the rest of the wells. Then, a 25 μL volume of the required concentration of (WP extract, MAU-EW, MAU-ME and MAU-AW) was added, and the plate was incubated for 48 h. At the end of incubation, a volume of (100 μL/well) containing two mixed reagents: glycyl-phenylalanylamino fluorocoumarin (GF-AFC, live-cell substrate) and [bisalanyl-alanyl-phenylalanyl-rhodamine 110 (bis-AAF-R110, dead-cell substrate), was added to all wells, mixed briefly on an orbital shaker and incubated for 30 min at 37 °C. The fluorescence was measured by the GloMax^®^-Multi Detection System for viability (excitation 495 nm; emission 505 nm), and (cytotoxicity: excitation 510 nm; emission 570 nm).

### 4.11. MALDI-TOF-MS Analysis

To obtain structural information, a MALDI- TOF-MS (Comstock Inc., Oak Ridge, TN, USA) analysis was performed using the LAZARUS II (home built), N_2_-laser (LSI VSL337ND) 337 nm, a 3-ns pulse width, focus diameter 0.1 mm, 16-kV acceleration voltage, 1-m drift length, data logging with LeCroy9450A, 2.5-ns sampling time and expected mass accuracy ±0.1%. Before the analysis, each sample was deposited from a solution in acetonitrile (ACN)/H_2_O (50/50) on a thin layer of 2,5-dihydroxybenzoic acid (DHB) crystals.

### 4.12. Statistical Analysis

The data were analyzed by one-way ANOVA followed by Tukey’s test (95% confidence) for multiple comparisons using SPSS (Version 20.0, IBM Corporation, New York, NY, USA). A *p*-value of <0.05 is considered statistically significant.

## Figures and Tables

**Table 1:** Effect of different concentrations of EJ extracts on IFN-γ production from unstimulated, phytohemagglutinin (PHA) + LPS stimulated mouse spleen cells, unstimulated T cells and NK cells and IL-12 production from unstimulated macrophages.

Extract	Concentration	Fold Change in IFN-γ				Fold Change in IL-12 from Unstimulated Macrophages
		Mouse Spleen Cells		Isolated Mouse Spleen Cells		
		Unstimulated	PHA + LPS Stimulated	Unstimulated T Cells	Unstimulated NK Cells	
WP	1 μg/mL	1.4	1.5	2.3 **	0.9	1.0
	10 μg/mL	1.7 **	2.7 ***	2.5 **	1.0	1.6
	100 μg/mL	2.0 **	4.2 ***	4.0 **	1.3	1.2
MAU	1 μg/mL	1.8 **	2.4 **	1.7 **	1.7 *	0.8
	10 μg/mL	2.4 **	3.9 ***	2.5 **	1.6 *	1.4
	100 μg/mL	3.2 **	5.7 ***	3.2 **	1.0	0.7
MAL	1 μg/mL	1.9 *	4.8 ***	2.1 *	0.8	1.1
	10 μg/mL	1.8 *	5.1 ***	0.6	0.9	1.5
	100 μg/mL	1.7	4.1 **	1.2	1.8 *	1.4

Each experiment was repeated 3 times with 5 replicates for each condition (* *p* < 0.05, ** *p* < 0.01, *** *p* < 0.001, compared to the 0 counterpart; fold change = value of cytokine production from cells with the extract/value of cytokine production from cells alone).

**Table 2:** Effect of different concentrations of sub-fractions from WP on IFN-γ and IL-12 production from stimulated and unstimulated mouse spleen cells.

Sub-Fraction	Concentration	Fold Change in IFN-γ		Fold Change in IL-12	
		Mouse Spleen Cells		Mouse Spleen Cells	
		Unstimulated	PHA + LPS	Unstimulated	PHA + LPS
WP	1 μg/mL	1.5	1.2	1.0	2.0
	10 μg/mL	2.4 *	1.9 *	1.7	2.2 *
MAU-EW	1 μg/mL	2.5 *	3.2 ***	1.5	2.7 **
	10 μg/mL	2.7 *	3.3 ***	1.8	2.7 **
MAU-ME	1 μg/mL	3.8 *	2.5 *	1.4	2.4 *
	10 μg/mL	3.8 *	2.5 *	1.5	2.0
MAU-AW	1 μg/mL	5.0 **	3.1 *	1.4	2.2
	10 μg/mL	5.4 **	3.2 *	1.8**	2.4

Each experiment was repeated 3 times with 5 replicates for each condition (* *p* < 0.05, ** *p* < 0.01, *** *p* < 0.001, compared to the 0 counterpart; fold change = value of cytokine production from cells with the extract/value of cytokine production from cells alone).

**Table 3:** Tentative interpretation of MALDI-TOF MS peaks in the WE, WP, MAU-EW, MAU-ME and MAU-AW sub-fractions.

Mass (*m*/*z*)	*Eriobotrya japonica* Hydrophilic Extracts and Sub Fractions					References
	WE	WP	MAU-EW	MAU-ME	MAU-AW	
503 ± 2	Eriojaposide A		Eriojaposide A			[12]
507 ± 2			Ursolic acid derivative			[5,19]
518 ± 2				9-*O*-apiosyl (1–6) glucoside		[12,13]
576 ± 2	A-type dimeric procyanidin (+proton)					[12,20,21,22]
580 ± 2	Naringenin-8-C rhamnoglucoside	Naringenin-8-C rhamnoglucoside	Naringenin-8-C rhamnoglucoside	Naringenin-8-C rhamnoglucoside	Naringenin-8-C rhamnoglucoside	[12,23]
588 ± 2			A-type dimeric procyanidin + (proton)			[12,20,21,22]
598 ± 2	Quercetin 3- sambubioside	Quercetin 3- sambubioside	Quercetin 3- sambubioside	Quercetin 3- sambubioside	Quercetin 3- sambubioside	[23,24]
610 ± 2			Kaempferol 3-*O*-sophoroside			[12,23]
637 ± 2	Cinchonain glucoside + (sodium)	Cinchonain glucoside + (sodium)	Cinchonain glucoside + (sodium)	Cinchonain glucoside + (sodium)	Cinchonain glucoside + (sodium)	[12]
651 ± 2		3-*O*-coumaroyl tormentic acid		3-*O*-coumaroyl tormentic acid		[25]
676 ± 2					Nerolidol 3-O-rhamnopyranosyl glycopyranosides	[26,27,28]
757 ± 2	Kaempferol 3-*O*-rhamnosyl glucoside-7-*O*-rhaminoside	Kaempferol 3-*O*-rhamnosyl glucoside-7-*O*-rhaminoside				[23,24]
774 ± 2	Quercetin 3-*O*-glucosyl-rhamnosyl-glucoside	Quercetin 3-*O*-glucosyl-rhamnosyl-glucoside		Quercetin 3-*O*-glucosyl-rhamnosyl-glucoside	Quercetin 3-*O*-glucosyl-rhamnosyl-glucoside	[23,24]
867 ± 2		Procyanidin C-1 + (proton)			Procyanidin C-1 + (proton)	[12,20]
1029 ± 2	Trimeric procyanidin + (gallic acid + glucose + sodium)				Trimeric procyanidin + (gallic acid + glucose + sodium)	[12,20]
1158 ± 2				B-type tetrameric procyanidin or A-type type + (proton)		[12,20]
1175 ±2				A-type tetrameric procyanidin + (sodium)		[12,20]
1197 ± 2				B-type tetrameric procyanidin + (sodium) Or dimeric cinchonain including two catechin units		[12,20]
1354 ± 2	B-type tetrameric procyanidin or A-type type + (sodium + gallic or glucose)				B-type tetrameric procyanidin or A-type type + (sodium + gallic or glucose)	[12,20,21,22]

## Supplementary Materials

Supplementary materials can be accessed at: http://www.mdpi.com/1420-3049/21/6/722/s1.

## Abbreviations

*Eriobotrya japonica* (EJ); water extract (WE); butanol-water phase (WP); methanol:acetone fraction upper phase (MAU); methanol:acetone fraction lower phase (MAL); ethanol:water eluted (MAU-EW); methanol:ethanol eluted (MAU-ME); acetone:water eluted (MAU-AW).
