# Supplementary material for: Eriobotrya japonica Water Extract Characterization: An Inducer of Interferon-Gamma Production Mainly by the JAK-STAT Pathway
Source: Molecules. 2016 Jun 2;21(6):722. doi: 10.3390/molecules21060722 (PMC6273127; doi:10.3390/molecules21060722)
Supplement: Supplementary file 1 [file molecules-21-00722-s001.pdf]

Supplementary Materials: *Eriobotrya japonica* Water Extract Characterization: An Inducer of Interferon-Gamma Production Mainly by JAK-STAT Pathway

Khalid Z. Matalka, Nada A. Abdulridha, Mujtaba M. Badr, Kenza Mansoor, Nidal A. Qinna and Fadi Qadan

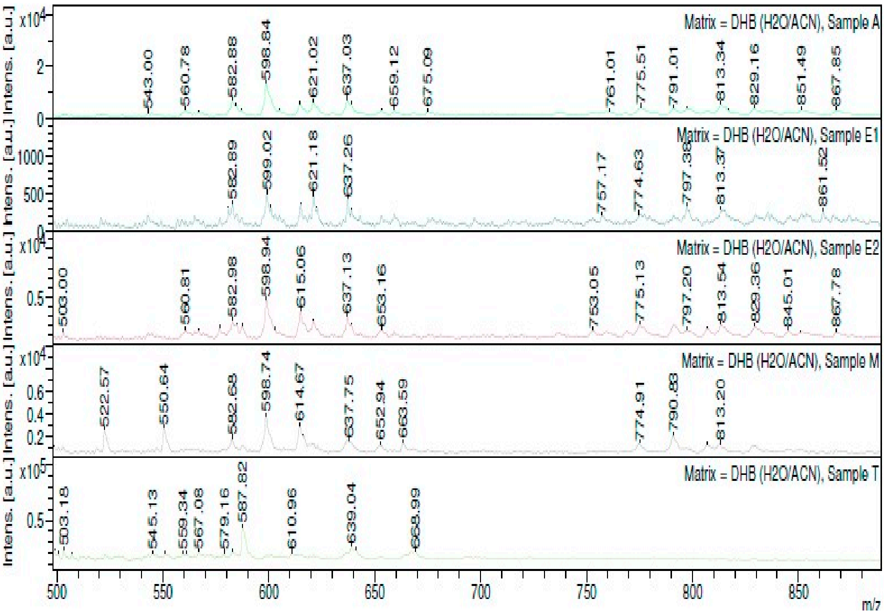

(**A**)

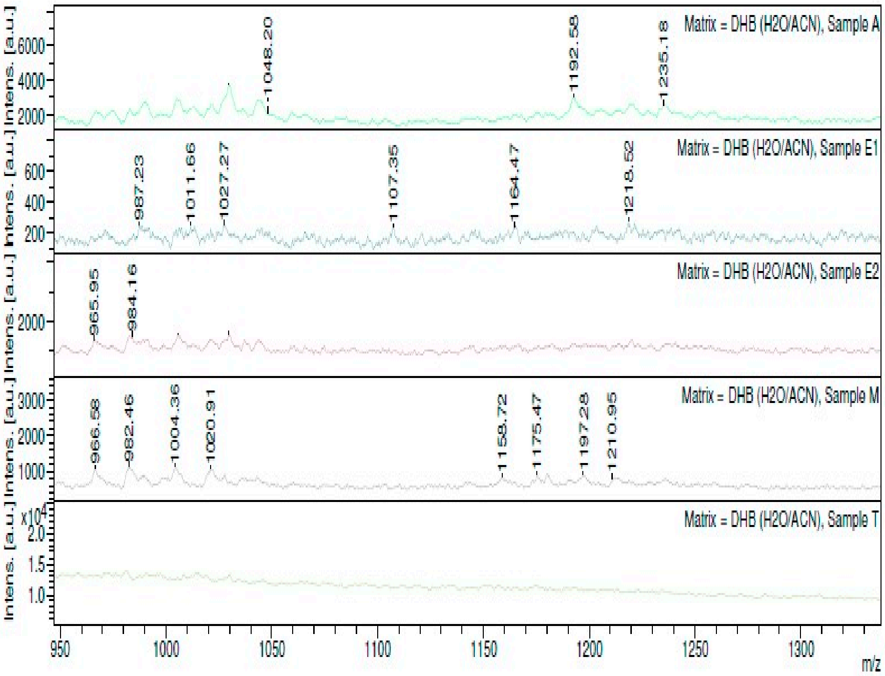

(**B**)

Figure S1. MALDI-TOF MS peaks in WE, WP, MAU-EW, MAU-ME and MAU-AW**.** The presented figures are examples of what was obtained from the MALD-TOF analysis. The above figures demonstrates the peaks obtained from *m*/*z* 500 to 890 (**A**) and from 900–1340 (**B**). Each of the extract or sub-fraction isolated form EJ leaves is denoted as E1: WE, E2: WP, A: MAU-AW, M: MAU-ME, and T: MAU-EW.
